# Physicians’ awareness of medication-related osteonecrosis of the jaw in patients with osteoporosis

**DOI:** 10.1371/journal.pone.0297500

**Published:** 2024-01-26

**Authors:** Nachapol Supanumpar, Pagaporn Pantuwadee Pisarnturakit, Natthinee Charatcharoenwitthaya, Keskanya Subbalekha

**Affiliations:** 1 Faculty of Dentistry, Department of Oral and Maxillofacial Surgery, Chulalongkorn University, Bangkok, Thailand; 2 Faculty of Dentistry, Department of Community Dentistry, Chulalongkorn University, Bangkok, Thailand; 3 Department of Medicine, Faculty of Medicine, Thammasat University, Pathum Thani, Thailand; Medical University of South Carolina, UNITED STATES

## Abstract

A serious adverse effect of antiresorptive drugs, which are widely used to treat osteoporosis, is medication-related osteonecrosis of the jaw (MRONJ). Physicians can reduce the risk of MRONJ by educating patients and emphasizing the importance of good oral health. However, limited information is available regarding physicians’ awareness and clinical practices associated with MRONJ. Hence, this study aimed to examine physicians’ awareness related to MRONJ and associated clinical practices. This study was a cross-sectional study conducted from December 2022 to February 2023. An online self-administered questionnaire was sent to physicians in Thailand who prescribed antiresorptive drugs for osteoporosis. Most respondents agreed that antiresorptive drugs might cause MRONJ (92.3%), poor oral health increased the risk of MRONJ (84%), and MRONJ is an important consideration in patients with osteoporosis (85%). Of the respondents, 48.1% and 15.5% always referred patients to dentists before and during antiresorptive therapy, respectively. Approximately 60% of physicians informed patients of the MRONJ risk before prescribing antiresorptive drugs, and 30% inquired about patients’ oral symptoms at the follow-up visit. Overall, 44% of physicians advised patients to receive oral health care; the most common reason for not advising this was that respondents did not consider themselves to be adequately knowledgeable to detect oral health problems. These findings indicate that while most physicians who prescribed antiresorptive drugs for osteoporosis were aware of and considered MRONJ in their practice, several took insufficient action to prevent it. This highlights the need to emphasize clinical practice guidelines and collaboration between physicians and dentists.

## Introduction

Osteoporosis is one of the most common metabolic bone diseases, with a global prevalence at 18.3%, and is most common in post-menopausal women and older men [1]. Since 1990, antiresorptive drugs such as bisphosphonates and denosumab have been widely used to treat osteoporosis in women and men [2,3]. These drugs are approved by the U.S. Food and Drug Administration and their ability to inhibit bone resorption is beneficial for treating osteoporosis and other conditions such as hypercalcemia of malignancy, Paget disease of the bone, and malignant metastases to the bone [4].

Despite these benefits in treating metabolic bone diseases, antiresorptive drugs have a major and severe adverse effect: medication-related osteonecrosis of the jaw (MRONJ) [5,6]. MRONJ is defined as necrotic bone exposure or bone that can be probed through an intraoral or extraoral fistula in the maxillofacial region for >8 weeks in patients treated previously or currently with antiresorptive drugs and with no history of radiation therapy [5]. Since MRONJ was first reported in 2003, the pathophysiological processes of the disease have not been fully clarified [7]. The treatment of MRONJ usually has unpredictable outcomes, and the disease affects numerous aspects of patients’ quality of life, including physical, mental, and psychosocial aspects. Moreover, MRONJ necessitates a long-term treatment and follow-up [5].

Among patients with osteoporosis who receive antiresorptive drugs, the incidence of MRONJ is 0.02%–0.3% [5,8]. To reduce this risk, dental risk factors must be minimized. This strategy is optimal when the focus is on regularly maintaining the oral health of patients at risk for MRONJ. Moreover, treatment in the early stages of MRONJ is more likely to be effective. Preventing MRONJ in patients with osteoporosis requires the cooperation of physicians and dentists. Physicians who are aware of MRONJ should explain its risk to patients, refer patients to dentists to remove possible sources of infection from the oral cavity to reduce the risk of MRONJ, and inquire regarding patients’ oral health status during follow-up visits [5,9–11]. To reduce the incidence and severity of MRONJ, preventive strategies should be used before, during, and after antiresorptive therapy. Dentists and physicians must collaborate to assess the risk factors that can lead to the development of MRONJ and devise a strategy to reduce such risk factors. Both actions are necessary for maintaining oral health, reducing the severity of MRONJ, and detecting possible early signs of this disease [11].

Several studies have reported that physicians who prescribe antiresorptive drugs to prevent bone metastases in patients with cancer provide more dental referrals and are more aware of MRONJ than physicians who prescribe these drugs to treat osteoporosis [12–14]. In several countries, few physicians provide dental referrals, indicating that few physicians are aware of MRONJ [12–16]. With the increase in the global older population, the incidence of osteoporosis and use of antiresorptive drugs also increase, and the incidence of MRONJ is expected to rise as well [17]. However, physicians’ awareness of MRONJ in patients with osteoporosis and the number of dental referrals are scarcely reported. Therefore, this study aimed to investigate awareness regarding MRONJ among physicians who prescribe antiresorptive drugs as well as the current multidisciplinary prevention and management of MRONJ in patients with osteoporosis.

## Materials and methods

In this cross-sectional study, we surveyed medical specialists, residents, and fellows in Thailand who prescribe antiresorptive drugs (e.g., in the fields of internal medicine, orthopedics, family medicine, gynecology and obstetrics, physical therapy and rehabilitation, and geriatric medicine) from December 2022 to February 2023. All procedures performed in this study involving human participants were in accordance with the ethical standards of the ethical committee of the Faculty of Dentistry, Chulalongkorn University, Bangkok, Thailand (approval no. HREC-DCU 2022–079). For the study to have a power of >95% and a significance level of 0.05 (two-sided test), the necessary number of participants was determined to be 125 [13]. We emailed an online self-administered questionnaire with a cover letter explaining the purpose of the study via a Google Form link and QR code to members of medical societies (the Thai Osteoporosis Foundation [TOPF], Endocrine Society of Thailand, Royal College of Physiatrists of Thailand, Royal College of Orthopaedic Surgeons of Thailand, Thai Society of Gerontology and geriatric medicine, Thai Rheumatism Association, and Thai Menopause Society).

“Bisphosphonate-related osteonecrosis of the jaw,” “antiresorptive agent–related osteonecrosis of the jaw,” and “osteonecrosis of the jaw” in general were defined as MRONJ to avoid confusion when respondents filled the questionnaire. The survey included 21 questions about demographics (11), awareness of MRONJ (3), related practices (6), and the role of physicians in reducing the risk of MRONJ (1; multiple answers). The content validity of the questionnaire was evaluated by three experts (an oral and maxillofacial surgeon, a dentist specializing in dental epidemiology, and an internal medicine physician) separately; moreover, the proposal and questionnaire were reviewed by physicians who are members of TOPF. The questionnaire was adjusted according to their recommendation. To test the reliability of the questionnaire, we conducted a pilot study including 10 physicians. Some confusing questions were adjusted for clarity. The first section of the questionnaire was regarding the physicians’ demographic characteristics, work sector, specialty, years of experience, frequency of prescribing antiresorptive drugs, most common antiresorptive drug prescribed, and experience with MRONJ. The second section of the questionnaire was concerning the physicians’ awareness of MRONJ and their practices in terms of patients’ oral health, such as providing dental referrals.

For data analysis, SPSS Statistics 28 (IBM Corporation, Armonk, NY, USA) was employed. Chi-square test and multivariable logistic regression were employed for bivariable and multivariable analyses. A *p* value of <0.05 was considered statistically significant.

## Results

### Demographic data

In total, 205 physicians responded to the questionnaire, of which 195 were included in the analysis as the other 10 did not prescribe antiresorptive drugs. The number of male and female respondents were 103 (52.8%) and 92 (47.2%). Overall, 44.1% of the respondents were in the age range of 30–34 years, and approximately half the respondents were instructors in medical schools. The majority of respondents belonged to the fields of internal medicine and orthopedics. Most respondents (56.4%) had prescribed antiresorptive drugs for <5 years, and 66.2% prescribed these drugs for <10 patients/month. Of the 195 respondents, 181 (92.9%) were aware of MRONJ; however, only 38 (19.5%) had treated patients with MRONJ. The sources of knowledge about MRONJ included textbooks, instructors, academic meetings, and media (journals, papers, and articles; Table 1).

**Table 1:** Demographic characteristics of the respondents (*N* = 195).

Characteristic	Group	*n* (%)
Sex	Men	103 (52.8)
	Women	92 (47.2)
Age	25–30 years	57 (29.2)
	31–40 years	86 (44.1)
	41–50 years	27 (13.8)
	>50 years	25 (12.8)
Work sector	Medical school	102 (52.3)
	Quaternary care center	37 (19)
	Tertiary care center	35 (17.9)
	Private hospital	21 (10.8)
Position at medical school (*n* = 102)	Instructor	56 (54.9)
	Resident	25 (24.5)
	Fellow	21 (20.6)
Specialty	Internal medicine	66 (33.8)
	Orthopedics	65 (33.3)
	Family medicine	20 (10.3)
	Gynecology and obstetrics	18 (9.2)
	Physical therapy and rehabilitation	26 (13.3)
Length of antiresorptive drug prescription	<5 years	110 (56.4)
	5–10 years	44 (22.6)
	>10 years	41 (21)
Frequency of drug prescription	<10 cases/month	129 (66.2)
	10–30 cases/month	50 (25.6)
	>30 cases/month	16 (8.2)
Recognition of MRONJ	Yes	181 (92.8)
	No	14 (7.2)
Practice includes patients with MRONJ	Yes	38 (19.5)
	Never	157 (80.5)
Source of knowledge about MRONJ	Textbook	42 (21.5)
	Instructor	41 (21)
	Academic meeting	51 (26.2)
	Journal/paper/article	47 (24.1)
	Never known	14 (7.2)
Have read articles about MRONJ in the past 3 years	Yes	124 (63.6)
	No	57 (29.2)

*MRONJ*; medication-related osteonecrosis of the jaw.

### Awareness of MRONJ

Most respondents agreed that antiresorptive drugs may cause MRONJ (92.3%), poor oral health increased the risk of MRONJ (84%), and MRONJ is an important consideration in patients with osteoporosis (85%; Table 2).

**Table 2:** Assessment of physicians’ awareness of MRONJ and related practices (*N* = 181).

Question	*n* (%)		
	Agree	Disagree	Not sure
**Awareness**			
1: Do antiresorptive drugs cause the risk of developing MRONJ?	167 (92.3)	1 (0.6)	13 (7.2)
2: Are patients with poor oral health conditions at greater risk for developing MRONJ than those with good oral health conditions?	152 (84)	11 (6.1)	18 (9.9)
3: Is MRONJ an important consideration in patients with osteoporosis?	172 (95)	2 (1.1)	7 (3.9)
	Always	Sometimes	Never
**Practice**			
4: Did you inform the patients about the risks associated with MRONJ before antiresorptive therapy?	113 (62.4)	53 (29.3)	15 (8.3)
5: Did you refer the patients to a dentist for an oral examination and preparation before antiresorptive therapy?	87 (48.1)	64 (35.4)	30 (16.6)
6: Did you refer the patients to a dentist for oral health care during antiresorptive therapy?	28 (15.5)	98 (54.1)	55 (30.4)
7: Did you inquire about the patients’ oral symptoms while monitoring them?	59 (32.6)	93 (51.4)	29 (16)
8: When you suspected that one of your patients has MRONJ, did you refer the patient to a dentist?	175 (96.7)	3 (1.7)	3 (1.7)
9: Did you recommend oral health care to the patients who receive antiresorptive drugs?	80 (44.2)	56 (30.9)	45 (24.9)

*MRONJ;* medication-related osteonecrosis of the jaw.

### Practice

MRONJ-related practices of the 181 respondents who were aware of MRONJ are listed in Table 2, and the reasons for answering practice items negatively are listed in Table 3. Approximately 60% of the respondents informed patients of the risks associated with MRONJ before starting antiresorptive therapy (question 4). The main reason why physicians did not inform patients of these risks before antiresorptive therapy was that the incidence of MRONJ is very low among patients with osteoporosis. Approximately 30% physicians inquired about patients’ oral symptoms during antiresorptive therapy (question 7). The most common reason for not inquiring was that patients did not mention oral symptoms (*n* = 101). Patients taking antiresorptive drugs received advice about oral health care from 80 physicians (question 9). The reason why 101 physicians did not give such advice was that they did not consider themselves sufficiently knowledgeable to detect oral health problems.

**Table 3:** Reasons why physicians did not always refer patients to dentists.

Reasons	Number of responses
Why did you not inform the patients of the details of the risks associated with MRONJ before starting antiresorptive therapy?	
• The risk of developing MRONJ is very low in patients with osteoporosis.	60
• I do not think antiresorptive drugs cause MRONJ.	5
• I think it is a detail that is not important to patients.	2
• Other (e.g., I only inform cases with a high risk of MRONJ, limited time, the patient has received medication from another doctor previously, and the patient no longer has any teeth.)	6
Why did you not refer the patients to a dentist for an oral examination and preparation before starting antiresorptive therapy?	
• I refer only those patients who are considered at risk.	64
• I think it unnecessarily burdens the dentists.	14
• I do not think dentists are involved in osteoporosis treatment.	10
• Osteoporosis needs to be treated urgently, before the patient sees the dentist.	13
• The referral system is difficult to navigate.	18
• Patients are uncooperative.	7
• Patients have difficulty paying for dental treatment.	8
• Other (e.g., the queue to see the dentist is long and I inquire about the patient’s oral health before referring; if there are no issues, I do not refer to a dentist.)	3
Why did you not refer the patients to a dentist for oral health care during antiresorptive therapy?	
• I refer only those patients who are considered at risk.	80
• Patients already have a dentist whom they visit regularly.	37
• I think it unnecessarily burdens dentists.	27
• The referral system is difficult to navigate.	20
• I do not think dentists are involved in osteoporosis treatment.	16
• Patients have difficulty paying for dental treatment.	13
• Patients are uncooperative.	5
• Other (e.g., the patient underwent an oral health assessment before starting antiresorptive therapy, I forgot to refer the patient to a dentist, I do not know if a referral is necessary, and the patient has prepared their oral cavity well.)	13
Why did you not inquire about patients’ oral symptoms while monitoring them?	
• The patients did not mention oral symptoms at all and did not inquire further.	101
• I think that oral health is not related to osteoporosis treatment.	8
• I think that the patients are already taking good care of their oral health.	22
• Other (e.g., I only inform cases with a high risk of MRONJ, limited time, and I ask some patients when it’s close to the time for their next dose.)	7
Why will you not refer the patients to a dentist if you suspect that they have MRONJ?	
• I will refer such patients to another specialist.	4
• I do not think dentists are helpful in MRONJ management.	2
• I can manage MRONJ myself.	1
• I think the symptoms are still unclear and the patient’s symptoms need to be monitored first.	1
Why did you not recommend oral health care to the patients who were receiving antiresorptive drugs?	
• I do not consider myself adequately knowledgeable to detect oral health problems.	55
• I think that the patients are already taking good care of their oral health.	30
• I think that oral health is not related to osteoporosis treatment.	14
• I think it is not my duty to give advice about oral health.	6
• Other (e.g., limited time, I think the patient has a low risk of MRONJ, and I think the patient has received advice from a dentist already.)	9

*MRONJ*; medication-related osteonecrosis of the jaw.

These answers were from the respondents who answered “never” or “sometimes” in questions 4–9. The participants could select multiple answers.

Most respondents agreed that oral health is related to the risk of MRONJ. The proportions of physicians who always and did not always refer patients to a dentist for oral examination and preparation before starting antiresorptive therapy were similar (question 5). Only 15.5% physicians always referred patients to a dentist for oral health care during antiresorptive therapy (question 6). The main reason why physicians did not refer the patients to a dentist before and during antiresorptive therapy (questions 5 and 6) was that they referred only patients who were considered to be at risk for MRONJ. If patients were suspected of having MRONJ, 96.7% physicians would refer them to a dentist (question 8). Only 4.9% respondents reported that they always practiced all the activities mentioned in questions 4–9.

### Factors associated with practice

Multivariable analysis revealed that the age of the physician was a factor associated with providing information about the risks of MRONJ to patients before starting antiresorptive therapy (question 4; *p* = 0.033). However, when we compared the age of the reference group (aged 25–30 years; comprising physicians with the least experience in treating patients with osteoporosis) with the age of the other groups, we found no difference. Univariable analysis of responses to question 7 revealed that physicians’ experience with patients who had MRONJ (*p* = 0.029) and reading articles about MRONJ (*p* = 0.025) were associated with inquiry about patients’ oral symptoms during follow-ups (question 7), whereas multivariable analysis did not reveal the effect of independent variables. Multivariable analysis revealed that position in medical school was associated with advising patients about oral health care (question 9): instructors tended to give this advice 3.13 times more often than residents and fellows (*p* = 0.028). Moreover, physicians who read articles about MRONJ (question 9) tended to advise patients about oral health care 3.17 times more often than those who did not read such articles (*p* = 0.005).

Factors that affected the decision to refer patients to dentists before starting antiresorptive therapy (question 5) included the physician’s specialty (*p* = 0.002) and period of antiresorptive drug prescription (*p* = 0.019). Physicians in the fields of internal medicine and family medicine tended to refer patients to dentists 6.02 times and 20 times more than those in the field of gynecologists and obstetricians, respectively. Physicians who had been prescribing antiresorptive drugs for <5 years were 3.5 times more likely to refer patients to dentists before starting antiresorptive therapy than those who had been prescribing such drugs for 5–10 years (*p* = 0.012). Referring patients to dentists after starting antiresorptive therapy was associated with physicians’ specialties (question 6; *p* = 0.03); however, comparing individual specialties with the reference specialty (gynecology and obstetrics, who were less involved in treating patients with osteoporosis compared with other specialties) revealed no differences. Physicians who had prescribed antiresorptive drugs for <5 years were 4.31 times more likely to refer patients to the dentist after starting antiresorptive therapy than those who had been prescribing such drugs for 5–10 years (*p* = 0.036). Physicians who had experience with patients with MRONJ were 50.92 times less likely to refer a patient with suspected MRONJ to a dentist (question 8) than those who had no experience with such patients (*p* = 0.021; Table 4).

**Table 4:** Multivariable analysis of factors associated with practice.

	Q4			Q5			Q6			Q7			Q8			Q9		
	AOR	95% CI	*p* value	AOR	95% CI	*p* value	AOR	95% CI	*p* value	AOR	95% CI	*p* value	AOR	95% CI	*p* value	AOR	95% CI	*p* value
Age																		
25–30 years ^ref^			0.033*			0.450			0.302			0.287			0.987			0.199
31–40 years	0.450	0.180–1.125	0.088	0.803	0.314–2.052	0.646	0.492	0.152–1.587	0.235	0.971	0.376–2.520	0.952	1.109	0.103–11.892	0.932	0.699	0.269–1.669	0.389
41–50 years	2.889	0.499–16.714	0.236	2.786	0.380–20.411	0.313	1.436	0.098–21.035	0.791	4.958	0.639–38.485	0.126	0	0	0.997	3.567	0.503–25.319	0.203
>50 years	1.272	0.220–7.351	0.788	3.858	0.459–32.417	0.214	5.589	0.216–144.327	0.300	5.544	0.679–45.299	0.110	0	0	0.996	3.993	0.529–30.149	0.179
Position in medical school																		
Student (resident and fellow)^ref^			0.772			0.713			0.192			0.136			0.893			0.092
Instructor	0.721	0.236–2.202	0.566	0.638	0.216–1.886	0.416	0.683	0.147–3.167	0.627	0.394	0.130–1.194	0.1	0.411	0.011–15.910	0.634	0.319	0.108–0.943	0.039*
Specialty																		
Gynecology and obstetrics ^ref^			0.167			0.002*			0.033*			0.606			0.879			0.572
Internal medicine	0.554	0.154–1.994	0.366	0.166	0.042–0.648	0.01*	0.145	0.016–1.334	0.088	0.609	0.151–2.463	0.487	0	0	0.995	0.397	0.102–1.552	0.184
Orthopedics	1.242	0.351–4.394	0.737	0.447	0.115–1.731	0.244	0.544	0.053–5.533	0.607	0.925	0.225–3.808	0.914	0.305	0.10–9.702	0.501	0.704	0.180–2.762	0.615
Family medicine	0.327	0.068–1.563	0.161	0.05	0.008–0.301	0.001*	0.162	0.15–1.774	0.136	0.622	0.114–3.407	0.584	0.555	0.025–12.362	0.710	0.476	0.095–2.394	0.368
Physical medicine and rehabilitation physicians	0.547	0.128–2.349	0.418	0.265	0.058–1.207	0.086	1.730	0.091–33.033	0.716	0.362	0.072–1.816	0.217	0.095	0.001–6.821	0.280	0.570	0.120–2.713	0.480
Length of antiresorptive drug prescription																		
<5 years ^ref^			0.852			0.019*			0.075			0.974			0.930			0.132
5−10 years	0.749	0.262–2.139	0.589	3.498	1.312–9.327	0.012*	4.311	1.096–16.950	0.036*	0.893	0.341–2.336	0.817	1.795	0.089–36.191	0.703	2.146	0.814–5.658	0.123
>10 years	0.696	0.131−3.701	0.671	0.692	0.98–4.882	0.711	0.677	0.47–9.675	0.773	0.904	0.129–6.313	0.919	14128675	0	0.996	0.569	0.085–3.794	0.560
Frequency of drug prescription																		
<10 cases/month ^ref^			0.360			0.244			0.622			0.286			0.920			0.724
10–30 cases/month	0.640	0.274–1.498	0.304	1.054	0.464–2.396	0.9	1.226	0.396–3.789	0.724	0.647	0.275–1.519	0.317	0.521	0.023–11.866	0.683	1.098	0.480–2.514	0.824
>30 cases/month	0.402	0.100–1.614	0199	3.748	0.759–18.508	0.105	3.374	0.290–39.310	0.332	0.319	0.072–1.402	0.130	0	0	0.998	0.634	0.158–2.543	0.520
Experience of patients with MRONJ																		
Yes	0.766	0.310–1.895	0.564	0.638	0.263–1.550	0.321	0.545	0.171–1.735	0.304	0.566	0.234–1.365	0.205	50.921	1.793–1446.275	0.021*	0.654	0.274–1.562	0.339
Never ^ref^																		
Read article about MRONJ																		
Yes	0.597	0.278–1.283	0.187	0.714	0.320–1.595	0.412	0.809	0.264–2.474	0.710	0.464	0.194–1.111	0.085	0.058	0.002–1.443	0.083	0.315	0.141–0.706	0.005*
No ^ref^																		

*MRONJ*; medication-related osteonecrosis of the jaw, *AOR*; adjusted odd ratio, *ref*; reference, *Q*; question, *CI*; confident interval

* *p* value < 0.05.

### Role in MRONJ prevention

To the question “What role do you think you play in reducing the risk of developing MRONJ?,” 115 physicians replied that they educated patients about and enhanced their understanding regarding antiresorptive drugs; for 110 physicians, education included explanations regarding the importance of oral health. In addition, 103 physicians thought that collaborating with a dentist helped reduce the risk of MRONJ.

## Discussion

We investigated the knowledge and awareness of Thai physicians regarding MRONJ and their related practice and found that almost all the physicians included in this study were aware of MRONJ (92.8%) and knew that MRONJ could occur in patients with osteoporosis (95%). This finding is the same as those reported by a Japanese study (94%) [15] and higher than that reported by a Brazilian study (78.66%) [14]. Conversely, only 31.5% physicians in Saudi Arabia [18] and 26.3% in Iraq [16] were aware of MRONJ. Among the physicians unaware of MRONJ, most worked in nonmedical schools compared with those who were aware of MRONJ, who were more likely to work in medical schools. This may be because information is always updated in medical schools. Most physicians who were unaware of MRONJ in this study were between the ages of 25 and 30 years. This may be because this group of doctors has the least experience in treating patients. However, <5% physicians in this study always informed their patients about MRONJ, referred patients to dentists, and considered patients’ oral health. These findings implied that most physicians know the adverse effects of the medications that they prescribe to their patients; they agree that oral health is related to the risk of MRONJ and that MRONJ is a serious condition of concern in patients with osteoporosis. In practice, however, it may not be possible to follow the 2022 clinical practice guidelines recommended by the American Association of Oral and Maxillofacial Surgeons (AAOMS) [5], which emphasize the importance of a multidisciplinary approach for treating patients receiving antiresorptive therapy, informing patients of the risk of MRONJ from antiresorptive therapy, and referring patients to dentists to remove possible sources of infection in the oral cavity and reduce the risk of MRONJ.

Previous studies have reported that maintaining good oral hygiene is most important in patients who require treatment with antiresorptive drugs; therefore, informing them of the dental risk of MRONJ and obtaining dental treatment before and after drug administration are crucial [19,20]. Several authors have recommended the extraction of teeth with poor prognoses before antiresorptive therapy to prevent MRONJ [21]. In addition, if all dental procedures are performed before antiresorptive therapy, future dentoalveolar surgery will not be required. A preventive strategy for proper oral health can reduce the incidence of MRONJ [5]. However, according to this study, the proportion of patients who received dental referrals before and during the administration of antiresorptive drugs was <50%. This finding is consistent with the data of many countries, such as Korea (<30%) [13], Japan (30%) [15], Brazil (17.99%) [14], India (49.2%) [22], and Iraq (15.8%) [16].

Herein, some physicians did not provide dental referrals except for patients considered to be at risk for MRONJ. This may lead to misdiagnosis or undertreatment in some cases; in a few patients with MRONJ, signs and symptoms can indicate subclinical diseases. Hence, before antiresorptive therapy begins, physicians should schedule dental consultations and dental follow-up for oral hygiene maintenance after patients start therapy. Additionally, as a result of physicians’ belief that referring a patient to a dentist is too burdensome for dentists, a patient’s oral health may be unprepared for antiresorptive medication, which in turn increases the likelihood that future surgery will be necessary and may increase the risk of MRONJ. Because MRONJ is an unpredictable and long-term condition and its incidence is increasing, the resulting burden on both physicians and dentists can compromise patient care.

Many physicians did not inquire about patients’ oral symptoms during follow-up because the patients did not mention oral symptoms, and many physicians did not advise their patients about oral health care because they were not knowledgeable about it. Dental referral is important because oral health care education, oral examination for early detection, and oral hygiene maintenance are important to reduce the risk of MRONJ. Physicians should at least mention the importance of oral health to patients and should inquire about patients’ oral health, using questions specifically about symptoms in the oral cavity.

The referral system should be improved for easy communication between physicians and dentists. Moreover, the oral care of patients with osteoporosis should be prioritized before starting antiresorptive therapy because some patients are at a high risk for fracture or because fracture has already occurred. The importance of dental examination and a well-coordinated referral system should be emphasized, as described in the clinical practice guidelines of the AAOMS [5] and TOPF [23]; physicians should be encouraged to adhere to those standards through open communication, collaboration with dentists, and routine provision of dental referrals to patients before and during antiresorptive therapy. Therefore, to decrease the risk and incidence of MRONJ, educational programs for physicians should include an emphasis on oral health and collaboration between professional healthcare providers.

A limitation of this study is that only 195 physicians responded to a questionnaire through a Google Form link and QR code; therefore, the probability of response bias should be considered. As there is no official registry regarding physicians who treat osteoporosis, the actual number of physicians who treat osteoporosis in Thailand remains unknown, and the sample in this study might not represent all physicians who prescribe antiresorptive drugs. Although we sent the questionnaire to medical associations, it might have not reached some physicians; therefore, we could not calculate the response rate. In further studies regarding the incidence of MRONJ, investigators should compare antiresorptive-treated patients who routinely maintain oral health with those who do not.

Most physicians who prescribe antiresorptive drugs are aware of and knowledgeable about MRONJ. In practice, however, it may not be possible to follow clinical practice guidelines strictly in certain circumstances. To improve the rate of dental referral, physicians should adhere to clinical practice guidelines and establish a routine of referring patients to dentists before and during antiresorptive therapy. To decrease the risk and incidence of MRONJ, educational programs for physicians should increase the awareness of oral health in patients with osteoporosis and emphasize collaboration between physicians and dentists.

## Supporting information

S1 File (XLSX)
